# Genetic variants in the *ITPR2* gene are associated with Kashin‐Beck Disease in Tibetan

**DOI:** 10.1002/mgg3.715

**Published:** 2019-05-07

**Authors:** Xue He, Mei Bai, Ming Liu, Li Wang, Yongjun He, Hao Rong, Dongya Yuan, Tianbo Jin

**Affiliations:** ^1^ Key Laboratory of Molecular Mechanism and Intervention Research for Plateau Diseases of Tibet Autonomous Region, School of Medicine Xizang Minzu University Xianyang China; ^2^ Key Laboratory of High Altitude Environment and Genes Related to Diseases of Tibet Autonomous Region, School of Medicine Xizang Minzu University Xianyang China; ^3^ Key Laboratory for Basic Life Science Research of Tibet Autonomous Region, School of Medicine Xizang Minzu University Xianyang China; ^4^ Department of gynaecology and obstetrics The Second Affiliated Hospital of Xi'an Jiaotong University Xi'an China; ^5^ Department of gynaecology and obstetrics The Ngari Prefecture People's Hospital Ngari Prefecture China

**Keywords:** case‐control study, *ITPR2*, Kashin‐Beck Disease, Single‐nucleotide polymorphisms, Tibetan

## Abstract

**Background:**

Kashin‐Beck Disease (KBD) is a chronic, endemic osteoarthropathy. Inositol 1,4,5‐triphosphate receptor type 2 **(** *ITPR2* **)** gene is involved in chondrocytes. We speculated that single‐nucleotide polymorphisms (SNPs) in *ITPR2* gene may have an association with KBD in Tibetan.

**Methods:**

To prove this hypothesis, a total of eight SNPs (rs1049376, rs11048526, rs11048556, rs11048585, rs16931011, rs10842759, rs2230372, and rs7134213) were selected, and genotyped in 316 KBD patients and 320 controls. The association between *ITPR2* variants and KBD risk was assessed by logistic regression analysis.

**Results:**

The study identified significant association (*p* = 0.019) between KBD and a novel locus, *ITPR2* SNP rs11048526 (OR = 1.49, 95% CI = 1.07–2.08). The variant A/G genotype frequency in rs11048526 had a significantly increased risk of KBD in co‐dominant model (OR = 3.70, 95% CI = 1.26–10.89, *p* = 0.018), dominant model (OR = 3.56, 95% CI = 1.22–10.40, *p* = 0.020), log‐additive model (OR = 3.00, 95% CI = 1.12–8.00, *p* = 0.029) after adjusted for age and gender.

**Conclusion:**

The results indicate a potential association between *ITPR2* and KBD risk in Tibetan. Further work is required to confirm these results and explore the mechanisms of these effects.

**Abbreviations**
CI: confidence interval
ER: endoplasmic reticulum
GWAS: genome‐wide association study
HWE: Hardy–Weinberg equilibrium
*ITPR2*: Inositol 1,4,5‐triphosphate receptor type 2
KBD: Kashin‐Beck Disease
OA: osteoarthritis
OR: odds ratios
RCC: renal cell carcinoma
SNP: single‐nucleotide polymorphism
WHR: waist‐to‐hip ratio

## 1 BACKGROUND

Kashin‐Beck Disease (KBD) is a chronic, endemic osteoarthropathy, which manifests as joint deformities and growth retardation (Hao et al., 2016, 2017). China is the country with the largest incidence of KBD in the world, and the Tibetans are the people most affected by KBD in China (Tai et al., 2017)**.** According to incomplete statistics, in 2013, there were 0.64 million patients with KBD and 1.16 million at risk in 377 counties of 13 provinces or autonomous regions in China (Guo et al., 2014). An endemic selenium deficiency, a serious cereal contamination by mycotoxin producing fungi and high humic acid levels in drinking water may be the major environmental causes of KBD (Duan et al., 2010). Based on the severity of joint lesions, KBD is clinically classified into three grades. Comparing with grade I, grade II, and III KBD patients have significant skeletal growth and development failure, such as shortened fingers, shortened humeri, and short stature (Hao et al., 2017).

Recent epidemiological and genetic study results suggest the interaction between environment factors and susceptibility genes might play a role in the KBD (Shi, Zhang, Lv, Wen, & Guo, 2015). The pathogenesis of KBD remains elusive (Zhang et al., 2015). Zhang et al. selected 559 patients with KBD and 467 healthy control subjects to identify susceptibility genes for KBD by conducting a two‐stage genome‐wide association study (GWAS), and identified that *ITPR2* (Inositol 1,4,5‐triphosphate receptor type 2; OMIM: 600144) effected the susceptibility of KBD in Han Chinese. (Zhang et al., 2015). Tai et al. selected the single‐nucleotide polymorphisms (SNPs) of 14 genes which composed of 849 KBD patients and 565 normal controls for association study in a Tibetan population, they found rs7775 in the *FRZB* gene may increase susceptibility to KBD, while rs7033979 in the *ASPN* gene may play a protective role in susceptibility to KBD in Tibetans. Moreover, genetic variants in genes *COL10A1* and *HABP2* may play a role in the risk of developing KBD in the Tibetan population (Tai et al., 2017). Huang et al. investigate the association of the selenoprotein genes *GPX1*, *TrxR2*, and *DIO2* with KBD in a Tibetan population. They found single SNPs in the genes *GPX1*, *TrxR2*, and *DIO2* may not be significantly associated with KBD in a Tibetan population (Huang et al., 2013). Several other susceptibility genes have been identified for KBD, including *HLA‐DRB1* (Shi et al., 2011), *GPX4* (Du et al., 2012), *GPX1* (Huang et al., 2013; Xiong et al., 2010), *ABI3BP* (Zhang et al., 2014), *SEPS1* (Du et al., 2015), *ITPR2* (Zhang et al., 2015), *ADAM12* (Hao et al., 2016), *COL2A1* (Hao et al., 2017), and so on. The genetic risk of KBD explained by reported susceptibility genes are limited, suggesting the existence of some new SNPs in the susceptibility genes *ITPR2* in Tibetan.

We selected eight SNPs (rs1049376, rs11048526, rs11048556, rs11048585, rs16931011, rs10842759, rs2230372, and rs7134213) of *ITPR2* for further genotyping, in order to understand the associations between the genetic variants of *ITPR2* and the susceptibility of KBD in Tibetan better. Our study might provide more significant evidence for further understanding of the KBD pathogenesis.

## 2 MATERIAL AND METHODS

### 2.1 Editorial policies and ethical considerations

The protocols for this study were approved by the Ethical Committee of the Xizang Minzu University, complied with the World Medical Association Declaration of Helsinki. All participants were informed both in writing and verbally to the procedures and purpose of the study and signed informed consent documents. All the subsequent research analyses were carried out in accordance with the approved guidelines and regulations.

### 2.2 Study participants

A total of 316 KBD patients and 320 healthy controls were consecutively recruited between June 2017 and July 2018 at the Ngari Prefecture People's Hospital, China. All of the subjects were recently diagnosed with KBD by undergoing careful clinical examination and radiography of the skeletal system. Patients with genetic bone and cartilage diseases, primary osteoarthritis (OA), rheumatoid arthritis, or a family history of articular disorders were excluded from the study. The controls were randomly selected as no KBD and no primary or secondary OA from the physical examination center of Ngari Prefecture People's Hospital. All control patients had no history of cancer.

### 2.3 SNP selection and genotyping

Using the database of 1,000 Genomes Project (http://www.1000genomes.org/) and dbSNP (https://www.ncbi.nlm.nih.gov/SNP/), candidate SNPs in the *ITPR2* gene with minor allele frequencies (MAFs)> 5% in the global population were selected. A total of eight SNPs (rs1049376, rs11048526, rs11048556, rs11048585, rs16931011, rs10842759, rs2230372, and rs7134213) were selected for further genotyping.

Peripheral blood samples (5 ml) were obtained from each participant. Genomic DNA was extracted from peripheral blood of cases and controls using the GoldMag‐Mini whole blood Genomic DNA Purification Kit (GoldMag Co. Ltd., Xi'an city, China), as recommended by the manufacturer's instructions (Geng et al., 2015). DNA concentration was determined by the NanoDrop Lite spectrophotometer (Thermo Fisher Scientific, Waltham, MA) (Wang et al., 2015). MassARRAY Nanodispenser (Agena Bioscience, San Diego, CA) was used to design primers for amplification process and single base extension reactions (Jin et al., 2015). SNP genotyping was carried out on the MassARRAY iPLEX (Agena Bioscience, SanDiego, CA) platform. Agena Bioscience Typer 4.0 software was used to manage and analyze SNP genotypic data.

### 2.4 Statistical analysis

Statistical analyses were performed using Microsoft Excel, SPSS 16.0 (SPSS, Chicago, IL) and PLINK 1.07 software. Deviation from Hardy–Weinberg equilibrium (HWE) was tested for control subjects to measure the distribution of the polymorphism using a Chi‐square test (Ren et al., 2016). Two‐side *p*‐value less than 0.05 were considered statistically significant. The risk associated with individual genotypes and allele was calculated as the odds ratios (OR) with their 95% confidence interval (95% CI) based on logistic regression models analysis (Bland & Altman, 2000). The Haploview software package (version 4.2) was used for analyses of linkage disequilibrium, haplotype construction (Barrett, Fry, Maller, & Daly, 2005; Shi & He, 2005). Associations between haplotypes and KBD risk were analyzed with PLINK. We used the unconditional logistic regression analysis adjusted for age and gender to determine the association between the haplotypes and KBD.

## 3 RESULTS

A cohort of 316 unrelated KBD patients with a mean age of 54.70 ± 17.14 years and 320 unrelated controls with a mean age of 19.05 ± 1.60 years involved in this case‐control study were presented in Table 1. There was a significant difference in age observed between the case and control groups (*p* < 0.0001).

**Table 1:** Characteristics of cases and controls in this study

Variable (s)	Case (*n* = 316)	Control (*n* = 320)	*p*
Sex (*N*, %)			<0.0001^a^
Male	183(57.9)	239(74.7)	
Female	133(42.1)	81(25.3)	
Age, years (mean ± *SD*)	54.70 ± 17.14	19.05 ± 1.60	<0.0001^b^

Note

*p* < 0.05 indicates statistical significance.

a Two‐sided chi‐squared test.

b Independent samples *t* test.

Table 2 shows the basic information and allele frequencies for each SNP in KBD patients and healthy controls. It is clear that information obtained from the Table 2 with regard to the SNPs and their chromosomal position, allele, minor allele frequency for cases and controls, and HWE test results. None of them were deviated from those expected by the HWE (*p* > 0.05), indicating good SNP genotyping quality. The minor allele frequency in the rs11048526 was 0.150 for cases compared with 0.106 for controls, which had a significant difference in allele frequency between cases and control groups; and the people carrier the “A” allele increased the risk of KBD compared with the individuals with the “G” allele in the allele model (OR = 1.49, 95% CI = 1.07–2.08, *p* = 0.019).

**Table 2:** Comparison of allele frequencies between cases and controls

SNP	Gene	Chromosome	Position	Alleles A/B	MAF		*p* value for HWE test	ORs (95% CI)	*p*
					Case	Control			
rs1049376	*ITPR2*	12	26,338,542	C/T	0.433	0.410	0.487	1.10 (0.88–1.37)	0.408
rs11048526	*ITPR2*	12	26,449,331	A/G	0.150	0.106	0.768	1.49 (1.07–2.08)	0.019*
rs11048556	*ITPR2*	12	26,515,735	A/G	0.351	0.302	0.894	1.26 (0.99–1.59)	0.058
rs11048585	*ITPR2*	12	26,568,249	T/C	0.296	0.284	0.272	1.06 (0.83–1.35)	0.651
rs16931011	*ITPR2*	12	26,575,582	G/A	0.108	0.092	0.740	1.19 (0.83–1.72)	0.350
rs10842759	*ITPR2*	12	26,613,060	A/G	0.417	0.422	0.567	0.98 (0.79–1.23)	0.873
rs2230372	*ITPR2*	12	26,631,917	A/G	0.448	0.450	0.910	0.99 (0.79–1.24)	0.937
rs7134213	*ITPR2*	12	26,666,946	T/C	0.359	0.353	0.807	1.03 (0.82–1.29)	0.822

Abbreviations: 95% CI, 95% confidence interval; HWE, Hardy–Weinberg equilibrium; MAF, minor allele frequency; OR, odds ratio; SNP, single‐nucleotide polymorphism.

* *p* < 0.05 indicates statistical significance.

Comparisons of the SNP genotypes and the risk of KBD under the genetic models were presented in Table 3. We observed the heterozygous variant A/G genotype frequency in *ITPR2* rs11048526 was significantly more in KBD patients than in healthy controls, and had an increased risk of KBD according to the co‐dominant model (OR = 3.70, 95% CI = 1.26–10.89, *p* = 0.018), dominant model (OR = 3.56, 95% CI = 1.22–10.40, *p* = 0.020), and log‐additive model (OR = 3.00, 95% CI = 1.12–8.00, *p* = 0.029) after adjusted for age. We also observed the heterozygous variant A/G genotype frequency in *ITPR2* rs11048556 was significantly less in KBD patients than in healthy controls, and had an increased risk of KBD according to the co‐dominant model (OR = 1.86, 95% CI = 1.10–3.13, *p* = 0.020), recessive model (OR = 1.84, 95% CI = 1.12–3.03, *p* = 0.016) before adjusted for age, but there was no association after adjustment analysis.

**Table 3:** Genotypic model analysis of the relationship between the SNPs and the risk of the KBD

SNP	Gene	Model	Genotype	Case	Control	Adjustment analysis		Crude analysis	
						OR (95% CI)^a^	*p* a	OR (95% CI)^b^	*p* b
rs11048526	*ITPR2*	Codominant	G/G	229	256	1		1	
			A/G	79	60	3.70 (1.26–10.89)	0.018*	1.47 (1.01–2.15)	0.046*
			A/A	8	4	0.86 (0.00–343.40)		2.24 (0.67–7.52)	
		Dominant	G/G	229	256	1		1	
			A/G‐A/A	27	64	3.56 (1.22–10.40)	0.020*	1.52 (1.05–2.20)	0.026*
		Recessive	G/G‐A/G	308	316	1		1	
			A/A	8	4	0.59 (0.00–213.70)	0.086	2.05 (0.61–6.88)	0.245
		Log‐additive	–	–	–	3.00 (1.12–8.00)	0.029*	1.48 (1.06–2.06)	0.021*
rs11048556	*ITPR2*	Codominant	G/G	140	155	1		1	
			A/G	126	137	1.30 (0.43–3.86)	0.642	1.02 (0.73–1.42)	
			A/A	47	28	4.10 (0.88–19.04)		1.86 (1.10–3.13)	0.020*
		Dominant	G/G	140	155	1		1	
			A/G‐A/A	173	165	1.64 (0.59–4.56)	0.345	1.16 (0.85–1.59)	0.35
		Recessive	G/G‐A/G	266	292	1		1	
			A/A	47	28	3.57 (0.87–14.64)	0.077	1.84 (1.12–3.03)	0.016*
		Log‐additive	–	–	–	1.82 (0.85–3.87)	0.123	1.24 (0.99–1.56)	0.066

Abbreviations: SNPs, single‐nucleotide polymorphisms; OR, odds ratio; 95% CI, 95% confidence interval.

a *p* values were calculated by logistic regression adjusted for age and gender.

b *p* values were calculated by logistic regression without adjustment for age and gender.

* *p* < 0.05 indicates statistical significance.

## 4 DISCUSSION

In this study, we performed eight new SNPs (rs1049376, rs11048526, rs11048556, rs11048585, rs16931011, rs10842759, rs2230372, and rs7134213) in the *ITPR2* gene associated with KBD in Tibetan. The SNP rs11048526 were significantly association with KBD which increased the KBD risk, our study results also support that the *ITPR2* is likely to be a susceptibility gene of the KBD.

Inositol 1,4,5‐triphosphate receptor (*IP_3_R*) type 2, is a protein which in human is encoded by the *ITPR2* gene, belonging to the IP_3_R family, which contains three isoforms. The protein encoded by this gene is a both a receptor for inositol triphosphate and a calcium channel. IP_3_Rs function as intracellular Ca^2+^ channels residing in the membrane of endoplasmic reticulum (ER). Through binding of IP_3_, IP_3_Rs mediate Ca^2+^ mobilization from ER to cytoplasm and play a key role in regulating physiologic processes. In vivo, IP_3_Rs have broad distribution in tissue and cell, including chondrocytes (Evans, Shen, Pollack, Aloia, & Yeh, 2005). As proapoptotic regulators, *IP_3_R* mediated calcium channels play an important role in maintaining intracellular Ca^2+^ homeostasis, which is essential for the control of apoptosis progression (Boehning, Patterson, & Snyder, 2004). Although the potential molecular mechanism of *ITPR2* involved in the development of KBD remains unclear, it is reasonable to speculate that *ITPR2* contributes to abnormal chondrocyte apoptosis in the articular cartilage of patients with KBD.

It was implication of the *ITPR2* in the KBD in Han Chinese has been reported, across the whole genome, one *ITPR2* SNP (rs10842750) was significantly associated with KBD, a suggestive evidence for association at *ITPR2* SNP was observed, significant associations between KBD and the seven SNPs selected from imputation analysis was also observed, including rs1531928, rs4414322, rs11048570, rs11048572, rs2017510, rs9669395, and rs1002835 (Zhang et al., 2015). In our study, the *ITPR2* also influences the occurrence of KBD in Tibetan. The allele “A” of rs11048526 in *ITPR2* had a significant destructive effect on KBD. Therefore, it is reasonable to infer that *ITPR2* gene played an important role in one's susceptibility to KBD who exposed to the same environmental. There were no more articles been reported about rs11048526 in the *ITPR2* gene with disease risk. About *ITPR2* gene with the other diseases risk association, some studies have been reported. Cecile et al. studies shown loci rs718314 near *ITPR2‐SSPN* exhibited marked sexual dimorphism which with a stronger effect on waist‐to‐hip ratio (WHR) in women than men(Heid et al., 2010). In another study, a locus near *ITPR2‐SSPN* (rs12814794) showed a nominal association with WHR in US Hispanic women (Graff et al., 2013). In an independent GWAS, Wu et al. identified two common variants rs718314 and rs1049380 in *ITPR2* gene as novel susceptibility loci for renal cell carcinoma (RCC; Wu et al., 2012).

There were some limitations in this study. It should be noted that there was a significant age difference between the KBD and the control samples in the case‐control study. To eliminate the potential impact of the age on the results, raw phenotypes were adjusted for the age as a covariate before the association analysis. The number of the selected SNPs was limited in genes *ITPR2* and the sample size was not large. Therefore the results need to be verified using well designed, high‐quality studies on well‐defined populations. Further studies are needed to confirm our finding and reveal the potential molecular mechanism of the *ITPR2* involve in the development of the KBD.

## 5 CONCLUSION

In summary, our study provided potential evidence that the genetic polymorphisms of *ITPR2* might contributed to the risk of KBD in the Tibetan. Next, we will further explore the molecular mechanism of *ITPR2* gene affecting the occurrence of KBD.

## 6 COMPETING INTERESTS

The authors declare that they have no conflict of interest.
